# Stabilising, diagnosing and preparing critically ill patients for transport

**DOI:** 10.1186/1757-7241-20-S2-P46

**Published:** 2012-04-16

**Authors:** Benjamin Ole Wolthers, Thomas Andersen Schmidt

**Affiliations:** 1 The Emergency Department, Holbaek Hospital, Denmark

## Background

The aim of this study was to investigate how many critically ill patients, initially hospitalized at the Emergency Department in Holbaek, get transferred for emergent treatment at tertiary centers. And especially to investigate how long it takes to stabilize, diagnose and prepare such patients for transport.

## Methods

Approximately 22,500 patients hospitalized at Holbaek Emergency Department from January 1. 2010 to June 23. 2011 were searched for patients discharged directly from the Emergency Department to another hospital. Afterward these patients had their medical journals revised for those patients needing acute transport with medical assistance.

The 32 cases found were divided into subgroups based on their diagnosis. The time from admittance to discharge was recorded as well as how old the patient was. An analysis of variance was performed to determine possible differences among the groups, both in age and admission time.

## Results

Thirty two patients were transferred to another hospital, amounting to 0.2% of the total number of patients referred for admittance in the Emergency Department.

Patients subgroups and their admission time(SEM):

• AMI(n:11): patients with STEMI based on ECG and chest pain- transferred to acute PCI. Hospitalized for 69.9 (8.58) minutes.

• Other(n:6): A heterogenic group including Third degree AV blok, Gastro-intestinal disorders, cramps and burn damages. Hospitalized for 82 (17.39) minutes.

• Vascular surgery(n:7): Arterial embolisms or aortic aneurysms. Transferred to acute surgery. Hospitalized for 108 (21.54) minutes.

• Neurosurgery(n:8): Intracranial haemorrhage, cauda equina or non stabile fractures of the spine. Transferred to acute surgery. Hospitalized for 141.8 (26.373) minutes.

## Conclusion

The time spent in the Emergency Department varies from 69.9 to 141.8 minutes among the subgroups. There is no significant difference in age, disregarding the “Other” group, which included 4 toddlers. The only significant time difference is between AMI patients and neurosurgery patients, i.e. neurosurgery patients requiring around twice as long(p < 0.05). The difference in time may be accounted for by the time needed to get a CAT-scan done- but is ~70 minutes acceptable? In Emergency Department settings more emphasis should be placed upon quickly evaluating patients radiographically and to prepare these acute patients for expeditious transport.

